# *Chlamydia pneumoniae*, heat shock proteins 60 and risk of secondary cardiovascular events in patients with coronary heart disease under special consideration of diabetes: a prospective study

**DOI:** 10.1186/1471-2261-6-17

**Published:** 2006-04-12

**Authors:** Mercy Guech-Ongey, Hermann Brenner, Dorothee Twardella, Dietrich Rothenbacher

**Affiliations:** 1 Department of Epidemiology, German Centre for Research on Ageing, University of Heidelberg, Heidelberg, Germany

## Abstract

**Background:**

There have been suggestions of an association between *Chlamydia pneumoniae*, chlamydial heat shock protein (Ch-hsp) 60 and human heat shock protein (h-hsp) 60 infection sero-status and development of secondary cardiovascular events. Patients with diabetes might be at higher risk since they are prone to infections. The objective of this study was to investigate prospectively the role of *Chlamydia pneumoniae *(CP), chlamydial heat shock protein (Ch-hsp) 60 and a possible intermediate role of human heat shock protein (h-hsp) 60 sero-status in the development of secondary cardiovascular disease (CVD) events in patients with coronary heart disease (CHD) under special consideration of diabetes mellitus.

**Methods:**

Patients aged 30–70 undergoing an in-patient rehabilitation program after acute manifestation of coronary heart disease (International Classification of Disease, 9^th ^Rev. pos. 410–414) between January 1999 and May 2000 in one of two participating rehabilitation clinics in Germany were included in this analysis. *Chlamydia pneumoniae *(CP), chlamydial heat shock protein (Ch-hsp) 60 and human heat shock protein (h-hsp) 60 status at baseline were measured by serum immunoglobulin G and A antibodies. Secondary CVD events (myocardial infarction, stroke, and cardiovascular death) were recorded during a mean follow-up period of 33.5 months (response = 87%).

**Results:**

Among the 1052 subjects 37.4% and 39.3% were sero-positive to CP IgA and IgG respectively, 22.2% were sero-positive to Ch-hsp 60 IgG and 8.4% were positive to h-hsp 60 IgG at baseline. During follow-up, secondary CVD events occurred among 71 (6.8%) participants. Occurrence of a secondary CVD event was more common among CP (IgA) and CP (IgG) sero-positive than among sero-negative patients (p-values 0.04 and 0.1, respectively). The risk of secondary CVD events was increased among patients with both a positive CP sero-status and diabetes compared to infection negative, non-diabetic patients and in general, sero-positivity added a hazard to diabetes. The interaction term between infection sero-status and diabetes was not statistically significant. We were not able to show an intermediate role of human heat shock protein (h-hsp) 60 sero-status in the development of secondary CVD events in patients with CHD.

**Conclusion:**

Results from this cohort of 1052 patients with pre-existing CHD cannot exclude a possible moderate increase in risk of secondary CVD events among patients with a positive infection sero-status. However, our study showed no intermediate role of human heat shock protein (h-hsp) 60 sero-status in the development of secondary CVD events in patients with CHD. Larger studies or meta-analysis of multiple studies are needed to address the interaction between infection sero-status and diabetes with adequate power.

## Background

*Chlamydia pneumoniae *(CP) is an obligate intracellular organism. Sero-positivity is low in early childhood (< 5 years), rising rapidly during school age such that at age 20 years approximately 50% of individuals will have detectable antibodies. Seroprevalence continues to rise, at a slower pace, in adulthood and reaches 75% in the elderly population [1]. The first description of the clinical presentation of *C. pneumoniae *infection appeared in 1985 [2].

*Chlamydia pneumoniae *is one infectious agent that has received particular attention as a potent atherogenic stimulus. Correlative studies suggest a possible relationship between atherosclerosis and CP infection [3,4]. Pathological studies have revealed the localization of CP in atherosclerotic lesions [5]. Several animal models have suggested a causal role of CP in atherogenesis [6,7], however, the precise mechanism(s) by which CP promote atherosclerosis still remains unclear.

Inflammation, infection and autoimmunity to human heat shock protein 60 (h-hsp60) have emerged as novel risk factors for coronary heart disease during the past decade. Heat shock proteins are a class of highly conserved proteins produced by all organisms as a result of stress and damage caused by, for instance, exposure to chemical and environmental factors, inflammation and infections [8]. Because of the high sequence homology between bacterial and human heat shock proteins, they have been postulated to be critical antigens in autoimmune diseases. In particular, it has been proposed that the immune response to microbial hsp60 may lead to autoimmunity to h-hsp60 and by that means nourish the development of atherosclerosis [9]. Because serum antibodies against chlamydial hsp60 from subjects with atherosclerosis may cross-react with human hsp60 and mediate endothelial cytotoxicity, [10] it is suggested that humoral immune reactions to hsp60s may play an important role in vascular endothelial injury, a key process in the early stages of atherosclerosis.

Two independent groups have shown that an elevated anti-human hsp60 antibody level is a risk factor for primary coronary atherosclerosis, especially when it is present with *Chlamydia pneumoniae *infection [11,12]. Therefore, it is suggested that heat shock protein (h-hsp) 60 may serve as a possible link between *Chlamydia pneumoniae *infection and atherosclerosis [12,13]. Elevated levels of h-hsp60 antibodies have also been reported in human sera obtained from patients with atherosclerosis [14]. In a recent study, Heltai and colleagues demonstrated associations of acute myocardial infarction with high levels of anti-h-hsp60 and anti-CP antibodies, these being independent risk factors for primary CHD [15].

The role of heat-shock proteins in patients with prevalent coronary heart disease on prognosis has been rarely investigated. Furthermore, despite the fact that patients with diabetes are immuno-compromised, remain persistently at risk for infectious complications and are prone to develop accelerated atherosclerosis [16], there are few epidemiological studies available investigating a potential interaction between chlamydial sero-markers of infection, heat shock proteins and secondary cardiovascular disease (CVD) events in diabetics.

In this study we are investigating prospectively the role of *Chlamydia pneumoniae*, chlamydial heat shock protein 60 and a possible intermediate role of human heat shock protein 60 sero-status in the development of secondary CVD events in patients with coronary heart disease (CHD) under special consideration of diabetes, also comparing the relationship between chlamydial sero-markers of infection, heat shock proteins and secondary cardiovascular disease (CVD) events in patients with diabetes with those without.

In addition, the association of CP sero-status and serum concentration of C-reactive protein (CRP) was explored, as CRP is considered a main determinant of secondary CVD events.

## Methods

### Study design and study population

All patients aged 30–70 years participating in an in-patient rehabilitation program after acute coronary syndrome (International Classification of Disease, 9^th ^Rev. pos. 410–414) or after coronary artery revascularisation between January 1999 and May 2000 in one of two participating rehabilitation clinics in Germany (Schwabenland-Klinik, Isny and Klinik am Südpark, Bad Nauheim) were enrolled in the study. Germany has a very comprehensive in-patient rehabilitation program and patients who were hospitalized due to acute coronary syndrome (i.e. unstable angina and myocardial infarction) or coronary artery revascularisation (i.e. coronary artery bypass grafting, coronary angioplasty, stent implantation) have the possibility to undergo an in-patient rehabilitation program (rehab) after discharge from the acute care hospital. The aims of this 3-weeks program are the reduction of cardiovascular disease risk factors, improvement of health related quality of life, and the preservation of the ability to work (latter only if a subject was working at onset of disease otherwise prevention of nursing care). This in-patient rehabilitation program begins approximately three weeks after the acute coronary syndrome or the revascularisation procedure but this time interval may be longer in some cases. In the current study only patients who were admitted within 3 months after the acute coronary syndrome or revascularisation procedure were included.

Follow-up was conducted one and three years after discharge from in-patient rehabilitation. All subjects gave written consent to the study. The study was approved by the Ethics Boards of the Universities of Ulm and Heidelberg and of the medical associations of the states of Baden-Wuerttemberg and Hessen.

### Data collection

At the beginning of the in-patient rehab all subjects filled out a standardized questionnaire containing sociodemographic information, smoking status and medical history, including a history of a physician's diagnosis of diabetes mellitus. In addition, information was taken from the patients' hospital charts, and a blood sample was drawn after overnight fast shortly before discharge.

Active follow-up was conducted one and three years after discharge from the rehab clinic. At both times information was obtained from patients via a mailed standardized questionnaire. A standardized questionnaire regarding cardiovascular disease events and treatment since discharge from the in-patient rehabilitation clinic was also obtained from the patient's primary care physician. If a subject died during follow-up, the death certificate was obtained from local Public Health authorities and the main cause of death was coded according to the International Classification of Diseases (9^th ^Revision).

Secondary cardiovascular disease (CVD) events were defined as CVD being main cause of death, or a physician's diagnosis of a non-fatal myocardial infarction or ischemic cerebrovascular event. For the analyses in this study, only subjects with sero-status of *Chlamydia pneumoniae *(CP), chlamydial heat shock protein (Ch-hsp) 60 and human heat shock protein (h-hsp) 60 and physician's questionnaire at follow up were included. Among 1206 patients with coronary heart disease recruited at baseline, follow-up information by physician's questionnaire or by death certificate could be obtained for 1052 patients (87%), of whom infection sero-status could be determined for 1030 (98%) patients for *Chlamydia pneumoniae*. H-hsp 60 was available in 983 (93%) patients.

### Laboratory analyses

Blood samples drawn before hospital discharge from the rehab clinic were prepared and serum for serology was stored at -80°C. Serum samples were analysed for *Chlamydia pneumoniae *by using specific enzyme immunoassay for the qualitative and semiquantitative detection of specific IgG or IgA antibodies (*Chlamdia pneumoniae*-IgG-sELISA medac and *Chlamdia pneumoniae*-IgA-sELISA medac, medac diagnostika, Hamburg, Germany).

The sero-positivity for the semiquantitative analysis was defined as cut-off index (sample optical density/cut-off) and evaluated as titer. Cut-off index ≤ 1.1 was defined as negative with titer < 1:50, cut-off index >1.1 and ≤ 1.8 was defined as positive with titer 1:50, cut-off index >1.8 and ≤ 3.6 was defined as positive with titer 1:100, cut-off index >3.6 and ≤ 7.2 was defined as positive with titer 1:200. Serum samples were also analysed by using recombinant enzyme immunoassays for chlamydial heat shock protein 60 (cHSP60-IgG-ELISA medac, medac diagnostika, Hamburg, Germany) and human heat shock protein 60 immunoglobulin G antibodies (hHSP60-IgG-ELISA medac, medac diagnostika, Hamburg, Germany). Serum samples were also analysed by using recombinant enzyme immunoassays for chlamydial heat shock protein 60 (cHSP60-IgG-ELISA medac, medac diagnostika, Hamburg, Germany) and human heat shock protein 60 immunoglobulin G antibodies (hHSP60-IgG-ELISA medac, medac diagnostika, Hamburg, Germany). Blood lipids were measured by routine methods and CRP was measured using a high sensitivity CRP (hsCRP) method (N latex CRP mono, Dade Behring, Marburg, Germany). All laboratory measures were done in blinded fashion.

### Statistical analyses

We first described the study population according to basic socioeconomic and medical characteristics. We then calculated the prevalence of sero-positivity to CP, Ch-hsp60 and h-hsp60 at baseline in the whole study population and in various age groups stratified for gender. We quantified possible associations with age by a chi-square statistic.

Furthermore, we described the incidence of secondary cardiovascular disease (CVD) events (cardiovascular death, myocardial infarction and stroke) and their combination during follow up. The occurrence of fatal and non-fatal CVD events during follow up according to sero-prevalence of CP, Ch-hsp60 and h-hsp60 at baseline was analysed by the life table method.

Finally, we used the Cox proportional hazards model to assess a possible association of sero-positivity to CP, Ch-hsp60 and h-hsp60 and diabetes (alone and in combination) at baseline with fatal and non-fatal CVD events during follow up while adjusting for the following covariates: age (years), gender (male, female), HDL-cholesterol (>40, ≤ 40 mg/dl), history of smoking (never, ever), alcohol consumption within last 12 months (none, any), school education (≤ 9 years, >9 years), marital status (married, other), history of myocardial infarction (never, ever), study centre (Bad Nauheim/Isny). Associations were quantified by hazard ratios (HR) and their 95% confidence interval (CI). In addition, a possible interaction between CP, Ch-hsp60 and h-hsp60 sero-status and diabetes was assessed by introducing a product term of each of the infection markers and history of diabetes in the adjusted model. In addition, age and gender adjusted geometric mean values of CRP (which is considered as a feature of atherosclerosis) were calculated and compared according to CP, Ch-hsp60 and h-hsp60 sero-status and history of diabetes by means of General Linear Regression methods. All analyses were carried out using the SAS software package, version 8.2.

## Results

Table 1 shows some basic characteristics of the entire study population with coronary heart disease and of patients with an additional history of diabetes (n = 199, 19.3%). Of the 1052 patients, 84.9% were male. The mean age of the study group was 60.0 ± 7.9 years and 56.9% of participants were in the age group 60–70 years. Almost all (97.8%) were of German nationality and 83.9% were married. About 58% reported a history of myocardial infarction. About 40% of the subjects had HDL-cholesterol levels ≥ 40 mg/dl and 17.6% of the entire study population had a body mass index > 30 kg/m^2 ^while 27.6% of patients with an additional history of diabetes had a body mass index > 30 kg/m^2^.

**Table 1:** Sociodemographic and medical characteristics of patients with coronary heart disease (CHD) (n = 1052) and an additional history of diabetes at baseline (n = 199)

**Characteristics**	**Total: N**	**%**	**Additional history of diabetes: N**	**%**
Gender				
Male	894	84.9	151	75.9
Female	158	15.0	48	24.1
**Age (years) (μ, SD)**	60.0 ± 7.9		60.9 ± 6.5	
30–49	148	14.1	15	7.5
50–59	305	29.0	50	25.2
60–70	599	56.9	134	67.3
Nationality a				
German	1028	97.8	195	98.0
Other	23	2.2	4	2.0
School education				
≤ 9 years	625	59.4	132	66.3
> 9 years	427	40.6	67	33.7
Family status				
Married	883	83.9	162	81.4
Other	169	16.1	37	18.6
Smoking				
Current	48	4.6	8	4.0
Former	667	63.4	120	60.3
Never	337	32.0	71	35.7
Alcohol consumption				
Any	790	75.1	131	65.8
None	262	24.9	68	34.2
**Body mass index (kg/m^2^)^b^**				
< 25	282	26.8	35	17.6
25–30	584	55.5	109	54.8
> 30	182	17.6	55	27.6
HDL-cholesterol (mg/dl)				
< 40	638	60.6	129	64.8
≥ 40	414	39.4	68	34.2
History of myocardial infarction	610	58.0	120	60.3

^a ^= There was one missing value for variable nationality

^b ^= There were four missing values for variable body mass index

Table 2 outlines sero-prevalence of *Chlamydia pneumoniae *(CP), chlamydial heat shock protein (ch-hsp) 60 and human heat shock protein (h-hsp) 60 sero-status at baseline in various age groups stratified by gender. Sero-positivity for CP IgA was 38.1% and 33.3% for males and females respectively and for CP IgG, it was 39.2% and 39.7% for males and females respectively. Sero-positivity for chlamydial heat shock protein (Ch-hsp) 60 (IgG) was 21.9% in males and 28.1% in females (overall 22.2%). Sero-positivity for human heat shock protein (h-hsp) 60 (IgG) was (8.7%) in males and (7.3%) in females (overall 8.4%).

**Table 2:** Sero-prevalence of *Chlamydia pneumoniae *and heat shock proteins 60 in the various age groups

	**Male N(%)**	**Female N(%)**	**All N(%)**
**N = 1030**	***Chlamydia pneumoniae *(IgA) positive**		

Age groups			
30–49	45 (34.4)	7 (46.7)	52 (35.6)
50–59	104 (39.4)	9 (25.0)	113 (37.7)
60–70	184 (38.3)	36 (34.3)	220 (37.7)
	p = 0.5	p = 0.8	P = 0.9

All	333 (38.1)	52 (33.3)	385 (37.4)

**N = 1030**	***Chlamydia pneumoniae *(IgG) positive**		

Age groups			
30–49	41 (31.5)	6 (40.0)	47 (32.4)
50–59	109 (41.1)	13 (36.1)	122 (40.5)
60–70	193 (40.3)	43 (41.0)	236 (40.4)
	p = 0.2	p = 0.9	p = 0.2
All	343 (39.2)	62 (39.7)	405 (39.3)

N = 1029	Chlamydia heat shock protein 60 positive		

Age groups			
30–49	26 (20.0)	2 (13.3)	28 (19.3)
50–59	52 (19.7)	7 (19.4)	59 (19.7)
60–70	114 (23.8)	27 (25.7)	141 (24.1)
	p = 0.4	p = 0.2	p = 0.2
All	192 (21.9)	36 (28.1)	228 (22.2)

**N = 983**	**Human heat shock protein 60 positive**		

Age groups			
30–49	13 (10.5)	1 (7.1)	14 (10.1)
50–59	22 (8.9)	5 (13.9)	27 (9.5)
60–70	37 (8.0)	5 (5.0)	42 (7.5)
	p = 0.4	p = 0.2	p = 0.5
All	72 (8.7)	11 (7.3)	83 (8.4)

Table 3 shows the occurrence of fatal and non-fatal cardiovascular disease events for the entire study population and for patients with and without a history of diabetes. During follow up (mean 33.5 months), 21 subjects suffered a cardiovascular death, another 30 a non-fatal myocardial infarction and 20 a non-fatal stroke, resulting in a total of 71 secondary CVD events. CVD events in general were higher in patients with an additional history of diabetes when compared to others.

**Table 3:** Cardiovascular disease (CVD) events during follow-up (mean 33.5 months) in patients with coronary heart disease at baseline and with respect to additional history of diabetes

**Cardiovascular (CVD) events during follow-up**	**N**			**%**		
	Total	DM	no DM	Total	DM	no DM

Cardiovascular death	21	12	9	2.0	6.0	1.1
Non fatal myocardial infarction	30	6	24	2.9	3.7	3.2
Non fatal Stroke	20	6	14	1.9	3.4	2.1

**Fatal and Non-Fatal events combined**	71	24	47	6.8	13.1	6.4

DM: Patients with a history of diabetes

no DM: Patients without a history of diabetes

Table 4 provides information on the occurrence of secondary CVD in relation to the sero-prevalence of CP, Ch-hsp60 and h-hsp60. Results from qualitative analyses showed that, for CP IgA, 8.8% sero-positive patients and 5.7% sero-negative patients had a secondary CVD event (p = 0.04). For CP IgG, 8.4% sero-positive patients and 5.9% sero-negative patients had a secondary CVD event (p = 0.1). In addition, results from quantitative analyses considering various levels of circulating antibodies of CP, Ch-hsp60 and h-hsp60 and secondary CVD events, antibody titers of < 1:50, 1:50–1:100 and ≥ 1:200 was not statistically significantly associated with secondary CVD events (results not shown).

**Table 4:** Occurrence of fatal and non-fatal CVD events during follow up according to sero-prevalence of *Chlamydia pneumoniae *(CP), chlamydia heat shock protein (ch-hsp)60, human heat shock protein (h-hsp)60 and diabetes (DM)

	**N (Column%)**	**Fatal and Non-Fatal CVD-Events During Follow-up N (column %)**	**P-value ^a^**
**CP – (IgA) (N = 1030)**			
negative	645 (62.6)	37 (5.7)	
positive	385 (37.4)	34 (8.8)	0.04
**Patients with DM (N = 199)**			
CP – (IgA)-negative	111 (55.8)	12 (10.8)	
CP – (IgA)-positive	88 (44.2)	12 (13.6)	0.5

**CP – (IgG)**			
negative	625 (60.7)	37 (5.9)	
positive	405 (39.3)	34 (8.4)	0.1
**Patients with DM (N = 199)**			
CP – (IgG)-negative	114 (57.3)	12 (10.5)	
CP – (IgG)-positive	85 (42.7)	12 (14.1)	0.4

**Ch-hsp60**			
negative	801 (77.8)	54 (6.7)	
positive	228 (22.2)	17 (7.5)	0.7
**Patients with DM (N = 199)**			
Ch-hsp60-negative	158 (79.4)	18 (11.4)	
Ch-hsp60-positive	41 (20.6)	6 (14.6)	0.6

**h-hsp60 (N = 983) ^b^**			
negative	900 (91.6)	64 (7.1)	
positive	83 (8.4)	4 (4.8)	0.4
**Patients with DM (N = 199)**			
h-hsp60-negative	186 (93.5)	21 (11.3)	
h-hsp60-positive	13 (6.5)	3 (23.1)	0.2

^a ^= Log Rank test used only on patients with measured antibody sero-status

^b ^= Only available in 983 CHD patients

Table 5 shows results of multivariate analyses using Cox proportional hazards model to assess the associations of sero-positivity to CP, Ch-hsp60 and h-hsp60 at baseline with secondary fatal and non-fatal CVD events during follow up. For the crude, partially adjusted (age and sex) and fully adjusted models, the hazard ratios (HR) for developing a secondary CVD event given a positive CP IgA titre at baseline were 1.60 (95% CI 1.01–2.55), 1.60 (95% CI 1.00–2.54) and 1.46 (95% CI 0.90–2.36) respectively, for CP IgG: 1.46 (95% CI 0.92–2.33), 1.44 (95% CI 0.91–2.30) and 1.39 (95% CI 0.87–2.23) respectively and for ch-hsp60 IgG titre at baseline were 1.12 (95% CI 0.65–1.93), 1.09 (95% CI 0.63–1.88) and 1.01 (95% CI 0.62–1.85) respectively. In the latter model, in addition to chlamydial heat shock protein, human heat shock protein sero-status was included but there was no change in the hazard ratio values (HR value for presence of h-hsp60 was 1.08 (95% CI 0.62–1.87) in the fully adjusted model. Furthermore, the interaction terms between CP IgA, CP IgG and Ch-hsp60 IgG sero-status and diabetes was not statistically significant given the limited number of events (p = 0.60, p = 0.86, p = 0.46) respectively. Furthermore, analyses from our quantitative data did not show any statistically significant association between levels of circulating antibodies of CP, Ch-hsp60 and h-hsp60 and secondary CVD events considering antibody titers of < 1:50, 1:50–1:100 and ≥ 1:200 (results not shown).

**Table 5:** Association of sero-positivity to *Chlamydia pneumoniae *(CP), chlamydia heat shock protein (ch-hsp)60 with secondary fatal and non-fatal CVD events during follow up: Results of a multivariate analysis

		**HR (95% CI)**		
		**Crude**	**Partially adjusted ^a^**	**Fully adjusted ^b^**
**CP (IgA)**				
	**Negative**	1^ref^	1^ref^	1^ref^
	**Positive**	1.60 (1.01–2.55)	1.60 (1.00–2.54)	1.46 (0.90–2.36)
**CP (IgG)**				
	**Negative**	1^ref^	1^ref^	1^ref^
	**Positive**	1.46 (0.92–2.33)	1.44 (0.91–2.30)	1.39 (0.87–2.23)
**ch-hsp60**				
	**Negative**	1^ref^	1^ref^	1^ref^
	**Positive**	1.12 (0.65–1.93)	1.09 (0.63–1.88)	1.01 (0.62–1.85)

^a ^= Adjusted for age and gender

b = Adjusted for age, gender, HDL-cholesterol, smoking, alcohol consumption, school education, marital status, history of myocardial infarction, study centre

(Test for interaction between diabetes and CP IgA (p = 0.60), CP IgG (p = 0.86) and ch-hsp60 (p = 0.46))

Table 6 shows in a multivariate analysis results of the association of combinations of sero-positivity to *Chlamydia pneumoniae *(CP), chlamydial heat shock protein (ch-hsp) 60 with history of diabetes and secondary CVD event. The risk of secondary CVD events was highest among patients with both a positive sero-status for CP IgA, CP IgG, Ch-hsp60 and diabetes and in general, sero-positivity tentatively increased the HRs of diabetes.

**Table 6:** Association of combinations of sero-positivity to *Chlamydia pneumoniae *(CP), chlamydia heat shock protein (ch-hsp)60 with history of diabetes and secondary fatal and non-fatal CVD events during follow up: Results of a multivariate analysis

	**HR (95% CI)**		
	**Crude**	**Partially adjusted ^a^**	**Fully adjusted ^b^**
**Combination of CP (IgA) and history of diabetes**			
CP (IgA) neg. and diabetes neg.	1^ref^	1^ref^	1^ref^
CP (IgA) neg. and diabetes pos.	2.41 (1.21–4.80)	2.31 (1.16–4.61)	2.16 (1.08–4.33)
CP (IgA) pos. and diabetes neg.	1.64 (0.92–2.90)	1.63 (0.93–2.90)	1.60 (0.89–2.87)
CP (IgA) pos. and diabetes pos.	3.20 (1.61–6.38)	3.04 (1.51–6.12)	2.63 (1.29–5.36)

**Combination of CP (IgG) and history of diabetes**			
CP (IgG) neg. and diabetes neg.	1^ref^	1^ref^	1^ref^
CP (IgG) neg. and diabetes pos.	2.21 (1.11–4.40)	2.10 (1.05–4.20)	1.88 (0.93–3.78)
CP (IgG) pos. and diabetes neg.	1.43 (0.81–2.53)	1.41 (0.79–2.50)	1.35 (0.76–2.41)
CP (IgG) pos. and diabetes pos.	3.21 (1.61–6.39)	3.07 (1.53–6.13)	2.77 (1.36–5.64)

**Combination of ch-hsp60 and history of diabetes**			
Ch-hsp60 neg. and diabetes neg.	1^ref^	1^ref^	1^ref^
Ch-hsp60 neg. and diabetes pos.	2.14 (1.22–3.78)	2.05 (1.15–3.63)	1.78 (1.01–3.18)
Ch-hsp60 pos. and diabetes neg.	1.06 (0.54–2.08)	1.04 (0.53–2.04)	0.93 (0.47–1.84)
Ch-hsp60 pos. and diabetes pos.	2.82 (1.19–6.68)	2.66 (1.12–6.35)	2.55 (1.06–6.17)

^a ^= Adjusted for age and gender

b = Adjusted for age, gender, HDL-cholesterol, smoking, alcohol consumption, school education, marital status, history of myocardial infarction, study centre

Furthermore, we found associations between CP IgA, CP IgG sero-positivity and CRP levels at baseline after adjusting for age and gender (geometric mean: CP IgA negative, 3.22 mg/L vs CP IgA positive, 3.98 mg/L, (p = 0.01)), (geometric mean: CP IgG negative, 3.31 mg/L vs CP IgG positive, 3.77 mg/L, (p = 0.07)). Our analyses also showed no association between Ch-hsp60 IgG and CRP levels at baseline after adjusting for age and gender.

## Discussion

In this prospective study, we investigated the role of and *Chlamydia pneumoniae *(CP), Chlamydia heat shock protein (Ch-hsp) 60 and a possible intermediate role of human heat shock protein (h-hsp) 60 antibodies in the development of secondary cardiovascular events in patients with coronary heart disease (CHD) under special consideration of diabetes mellitus. The results from this study showed that the occurrence of a secondary CVD event was more common among CP (IgA) and tentatively CP (IgG) sero-positive than among sero-negative patients in crude analysis. However, the associations and a possible interaction between the sero-status of these infections and history of diabetes were not statistically significant after multivariate adjustment for covariates. Although the study clearly demonstrated an increased risk in developing a secondary CVD event among sero-positive patients with a history of diabetes compared to sero-negative without and sero-positivity adding a hazard to diabetes, it is suggested that mainly diabetes was responsible for the increased risk. Furthermore, this study showed that human heat shock protein 60 (h-hsp60) is unlikely an intermediate factor for developing secondary CVD events in the population studied.

*Chlamydia pneumoniae *is one infectious agent that has received particular attention as a potent atherogenic stimulus. However, the question whether serological evidence of prior infection with CP and the presence of Ch-hsp60 and h-hsp60 is associated with MI and CHD death remains a source of controversy, as several prospective studies have failed to demonstrate consistent associations between the presence of IgG antibodies to CP and incident MI [17-19]. Examples of some studies that looked at primary CVD events include a nested case-control study conducted within the context of the Cardiovascular Health Study [20]. Results showed that high-titer CP IgG was associated with an increased risk of primary CHD (OR 2.2, 95% CI, 1.1–4.4), whereas, a low to moderate CP antibody titre showed a weak non-significant association of IgG antibodies to CP with the risk of primary CHD among the elderly (OR 1.1, 95% CI, 0.7 to 1.8). Furthermore, Haider and colleagues found CP infection, as evidenced by sero-positivity, not to be associated with increased risk for primary CVD in the Framingham Heart Study cohort: CP IgG (HR 0.91, 95% CI, 0.68–1.20) and CP IgA (HR 0.65, 95% CI, 0.39–1.07) [21].

Further examples of the limited number of studies that looked at CP sero-positivity and secondary CVD events include a prospective study in the context of the Heart Outcomes Prevention Evaluation (HOPE) Study. Their study showed that among patients with pre-existing CVD, neither CP IgG (adjusted HR, 0.87; 95% CI, 0.68–1.10) nor CP IgA (adjusted HR, 1.10; 95% CI, 0.90–1.34) predicted cardiovascular events during the 4.5 years of follow-up. [22]. Another prospective study evaluated the effect of pathogens and pathogen burden on the risk of MI or death among coronary artery disease (CAD) patients. This study demonstrated that baseline prevalence of antibodies directed against CP was not significantly associated with development of secondary MI or CVD death in an elderly cohort after a 3 years follow-up period. [19]. Furthermore, Haim and colleagues evaluated the association between previous exposure to CP and future coronary risk in patients with CHD. This was a prospective nested case-control design including patients from a trial study of bezafibrate for the treatment of CHD. Results showed that mean titers of anti-CP antibodies (IgG and IgA) in the baseline sera of 136 patients who had coronary events during a mean follow-up period of 6.2 years were similar with 136 age- and gender-matched controls from same trial without subsequent coronary events. [23].

In addition, another study investigated the role of infections, autoimmunity and inflammation in atherosclerosis. They studied the joint effect of chronic *Chlamydia pneumoniae *(CP) infection, persistently elevated human heat-shock protein 60 (h-hsp60) antibodies and C-reactive protein (CRP) on coronary risk. Results showed that, compared with persistently low levels, the risk of coronary events was 2-fold for persistently elevated IgA antibodies to CP (OR, 1.96; 95% CI, 1.14–3.36) and also for IgA antibodies to h-hsp60 (OR,2.11; 95% CI, 1.08–4.13). The risks associated with elevated antibodies were much higher when CRP was also elevated [24]. In a previous study, Huittinen and colleagues showed that an elevated level of IgA antibodies to h-hsp60 in baseline serum predicted the occurrence of coronary event several years later [11]. Although CP, a common respiratory pathogen throughout the world, has been associated with atherosclerosis, in the above mentioned study, this finding indicated that elevated levels of CP antibodies were not alone a significant risk factor for coronary events. Instead, a 7-fold risk emerged when an elevated level of IgA antibodies to CP in baseline serum was accompanied by factors related to auto-immunity and inflammation, such as elevated levels of h-hsp60 IgA antibodies and CRP. Although hsCRP levels were associated with *C. pneumoniae *antibodies in our study, elevated hsCRP levels of patients did not have any type of joint effect in predicting secondary cardiac event (data not shown). This is in accordance with observations of others [25,26]. CRP may be a more modest predictor of CHD in elderly subjects, especially if already having CHD, than previously reported. The intake of aspirin and statins may further attenuate the predictive power for later events in patients with CHD.

Furthermore, all patients included in this study had an acute cardiovascular event within last 3 months before baseline. However, such an event may also reduce antibody titers, as has been shown for (h-hsp) 60 [27] and this may partly explain the negative results for secondary events in patients with prevalent CHD of our study.

The following limitations of our study should be considered. Although we had a large sample of patients with CHD (over 50% with a MI), fatal-CVD events were rare in this study population. This might be explained by the fact that mortality of MI is highest within the pre- and early hospital phase. As the acute events leading to diagnosis of CHD or MI had occurred at least 3 weeks before inclusion in this study, we were dealing with a selection of patients with a better prognosis compared to a patient population within the early phase of a newly diagnosed CHD. Furthermore, not all patients of course are willing or able to participate in a rehab program. This is another reason why severely ill-patients are probably underrepresented in our study sample. In addition, proof of chlamydial heat shock protein 60 immunoglobulin G is genus specific and not specific to *C. pneumoniae*, although the majority of antibodies against chlamdial LPS are due to *C. pneumoniae*.

Although inflammation represents an important feature in all stages of atherogenesis and its complications [28,29], we could not demonstrate an association between infection markers, sero-status and levels of CRP, even after consideration of history of diabetes. This may indicate, however, that another pathway might be of more importance.

Despite the above mentioned limitations, results of our study cannot exclude a possible moderate increase in risk of secondary CVD events among patients with a positive infection sero-status, however, they clearly indicate an increased risk among diabetic patients. This study further showed that h-hsp60 is unlikely an intermediate factor for developing secondary CVD events considering mechanisms involved in autoimmunity. Larger studies or meta-analysis of multiple studies are needed to address the interaction between infection sero-status and diabetes with adequate power. Notwithstanding the need of further evidence from such studies, patients with diabetes are at special risk for developing secondary CVD events and should be a target group for preventive measures.

## Conclusion

Results from this cohort of 1052 patients with pre-existing CHD cannot exclude a possible moderate increase in risk of secondary CVD events among patients with a positive infection sero-status. However, our study showed no intermediate role of human heat shock protein (h-hsp) 60 sero-status in the development of secondary CVD events in patients with CHD. Larger studies or meta-analysis of multiple studies are needed to address the interaction between infection sero-status and diabetes with adequate power.

## Abbreviations

CI = confidence interval

HbA1c = hemoglobin A1c

OR = Odds ratio

## Competing interests

The author(s) declare that they have no competing interests.

## Authors' contribution

MGO did the statistical analysis and wrote the initial draft.

HB and DR had the idea of the study and HB, TD and DR did study design and conduct. All authors critically revised the MS and read and approved the final manuscript.

## Pre-publication history

The pre-publication history for this paper can be accessed here:


